# Mpox: disease manifestations and therapeutic development

**DOI:** 10.1128/jvi.00152-25

**Published:** 2025-08-11

**Authors:** Yining Wang, Xin Wang, Tunca Doğan, Nadia A. Sam-Agudu, Jaffar A. Al-Tawfiq, Qiuwei Pan

**Affiliations:** 1 Department of Gastroenterology and Hepatology, Erasmus MC University Medical Center 6993, Rotterdam, the Netherlands; 2 Department of Computer Engineering, Biological Data Science Lab, Hacettepe University 37515, Ankara, Turkey; 3 Department of Bioinformatics, Graduate School of Health Sciences, Hacettepe University 37515, Ankara, Turkey; 4 Department of Health Informatics, Institute of Informatics, Hacettepe University 37515, Ankara, Turkey; 5 International Research Center of Excellence, Institute of Human Virology Nigeria 603970, Abuja, Nigeria; 6 Department of Pediatrics and Child Health, School of Medical Sciences, University of Cape Coast 107841, Cape Coast, Ghana; 7 Global Pediatrics Program and Division of Infectious Diseases, Department of Pediatrics, University of Minnesota Medical School 12269, Minneapolis, Minnesota, USA; 8 Infectious Disease Unit, Specialty Internal Medicine, Johns Hopkins Aramco Healthcare, Dhahran, Saudi Arabia; 9 Division of Infectious Diseases, Indiana University School of Medicine 12250, Indianapolis, Indiana, USA; 10 Division of Infectious Diseases, Johns Hopkins University 1466, Baltimore, Maryland, USA; 11 Accreditation and Infection Control Division, Quality and Patient Safety Department, Johns Hopkins Aramco Healthcare, Dhahran, Saudi Arabia; Universiteit Gent, Merelbeke, Belgium

**Keywords:** monkeypox virus, clinical feature, experimental model, therapeutic development

## Abstract

Mpox, caused by monkeypox virus (MPXV) infection, has emerged as a significant global health threat. The World Health Organization (WHO) has twice declared a Public Health Emergency of International Concern for mpox: first for the 2022–2023 global outbreak and subsequently for concurrent outbreaks in Africa. Beyond MPXV, other members of the Orthopoxvirus genus also pose growing risks of zoonotic spillover, with the potential to jump from animal reservoirs to humans. Clinically, mpox is distinguished from other Orthopoxvirus infections by its propensity to cause severe systemic manifestations alongside localized skin lesions, disproportionately affecting vulnerable groups such as children, pregnant women, and immunocompromised individuals. Although vaccines are available, effective therapeutics are equally essential in combating the mpox crisis. Current antiviral agents, including tecovirimat and brincidofovir, have demonstrated uncertain or disappointing efficacy in preclinical and clinical studies, underscoring the urgent need for further therapeutic development. This review provides a concise synthesis of recent advances in understanding mpox epidemiology and clinical features and offers an in-depth discussion of the current status and future directions in therapeutic development. We highlight the importance of innovative experimental models that can authentically replicate mpox disease manifestations and serve as robust platforms for therapeutic testing. Advancing these research efforts is critical for responding to the ongoing mpox emergency and for sustaining preparedness against future poxvirus epidemics.

## INTRODUCTION

Since the first mpox case was reported in 1970 from the Democratic Republic of the Congo (DRC), monkeypox virus (MPXV) has been endemic in Africa, particularly in West and Central Africa (1). Genetically, MPXV is classified into two major clades: clade I (subclades Ia and Ib) and clade II (subclades IIa and IIb). The 2022–2023 clade IIb global outbreak resulted in the highest number of mpox cases in non-endemic regions, with most infections reported in Europe and the Americas (2, 3). This mpox outbreak primarily spread through sexual contact among men who have sex with men (4). The World Health Organization (WHO) for the first time designated mpox as a Public Health Emergency of International Concern (PHEIC). Since early 2025, Sierra Leone has experienced its largest recorded mpox outbreak to date, marking the first major epidemic in Africa caused by the clade IIb strain (5).

In August 2024, the emergence of the clade Ib strain, its swift spread in eastern DRC, and the reporting of cases in several neighboring countries in Africa prompted the declaration of mpox as a PHEIC once again (6). As the fastest-expanding strain, travel-related clade Ib cases have been reported in a list of countries (7–10). Genomic data suggested that clade Ib is transmitted exclusively through human-to-human contact, mirroring the 2022–2023 clade IIb outbreak (11). As the outbreak progresses, transmission dynamics are evolving, with increasing household spread leading to a shift in age and sex distribution—particularly a rise in pediatric cases (6).

Clade Ia, first identified in the DRC, remains endemic in several provinces of the country. Since 2023, sporadic cases have been reported in the Republic of the Congo and the Central African Republic, with a higher proportion of pediatric cases in the latter (12). Phylogenetic analyzes indicate that clade Ia outbreaks stem from zoonotic spillover followed by secondary human-to-human transmission. Additionally, sustained sexual transmission networks in the DRC may contribute to increasing case importation in endemic areas (13).

Clade IIa is the least characterized MPXV strain. Although infections have been documented in adults and children, limited epidemiological data hinder a full understanding of their transmission dynamics. In 2024, clade IIa cases were reported in Côte d’Ivoire, Guinea, and Liberia, marking the first evidence of sustained community transmission and co-circulation with clade IIb in these regions (14). While no confirmed sexual transmission has been reported, close contact likely facilitates its spread, as seen with other clades. Collectively, although sexual contact remains a major transmission route for the recent outbreaks, all MPXV clades can spread through non-sexual direct contacts, including household and community exposure, which pose a tremendous threat to the global population.

## MAIN CLINICAL FEATURES AND MANIFESTATIONS

### Distinct features

**Fig 1:**
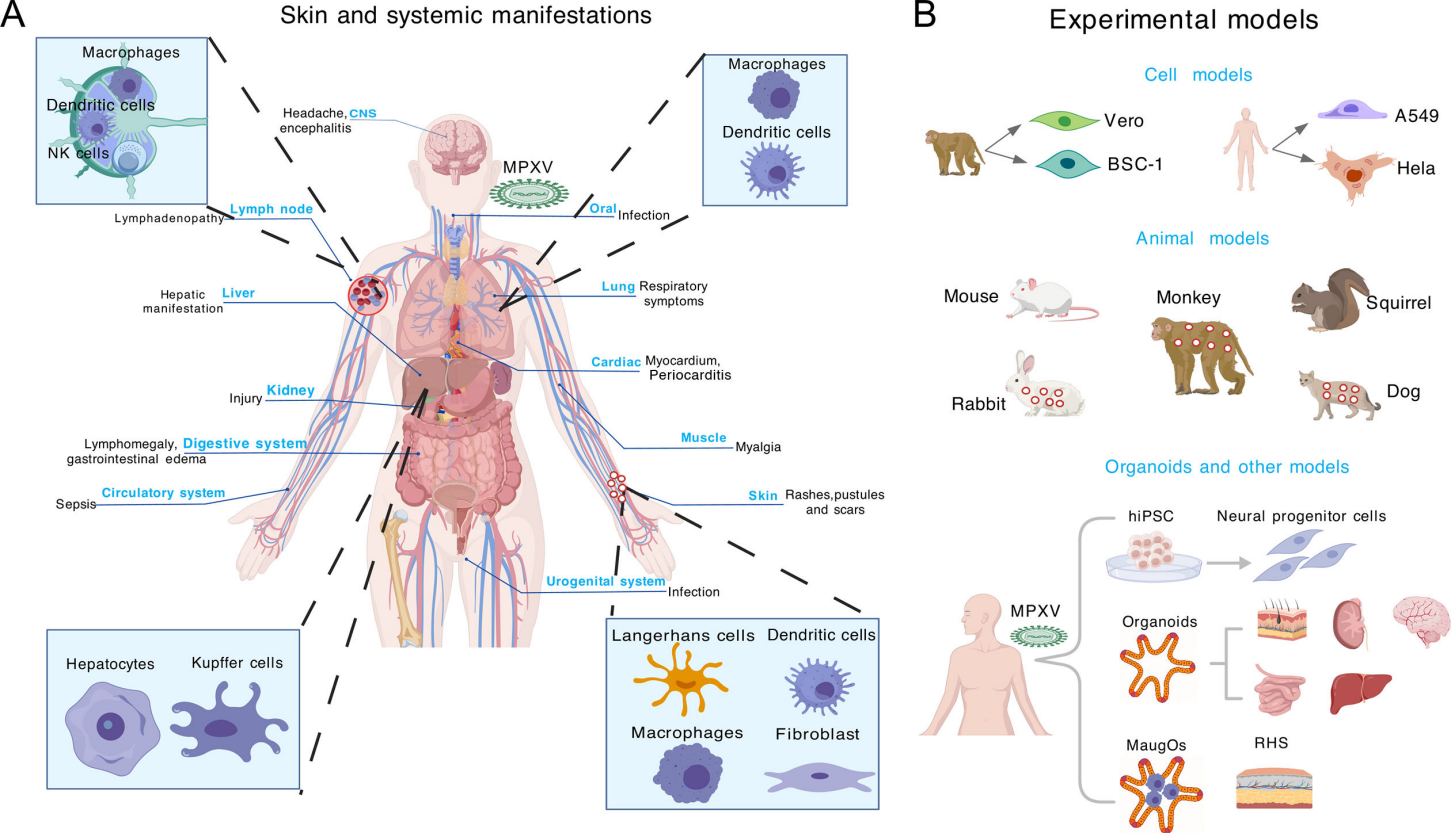
Clinical features, disease manifestations, and experimental models of mpox. (**A**) Skin and systemic manifestations of mpox. In addition to skin lesions, monkeypox virus (MPXV) infection can cause systemic symptoms and manifestations. Mpox-associated systemic manifestations include cardiovascular complications, neurological complications, kidney injury, proctitis (inflammation of the rectum), respiratory symptoms, digestive complications, circulatory manifestations, and pregnancy-related manifestations. MPXV transmission occurs mainly through respiratory and cutaneous routes. In the respiratory tract, the virus infects not only airway epithelial cells but also immune cells such as dendritic cells and macrophages. In the skin, MPXV infects keratinocytes and fibroblasts and may also infect skin-resident immune cells such as dendritic cells, macrophages, and Langerhans cells. A key feature of mpox is swelling of lymph nodes (lymphadenopathy), reflecting systemic inflammatory response, which may be caused by abnormal proliferation and retention of natural killer (NK) cells, and infected macrophages and dendritic cells. Following its spread through lymphoid tissue, MPXV may target other organs such as the liver. MPXV has been shown to infect hepatocytes and Kupffer cells in the liver. (**B**) Experimental models for MPXV infection. Immortalized cell lines originating from monkey and human sources have been widely used for MPVX infection studies *in vitro*. A number of animal models have been established to support MPXV infection and can be used for testing antiviral treatments. Among these models, monkeys, rabbits, and dogs can develop skin lesions upon MPXV infection. To study neurological complications, human-induced pluripotent stem cells (hiPSC)-derived neural progenitor cells have been used to model MPXV infection. To study the broad-spectrum tissue tropism and disease manifestations, a variety of organoids—including human skin, kidney, brain, intestine, and liver organoids generated from hiPSC or primary tissues—have been employed to model MPXV infection. A recent study has demonstrated the proof of concept of simultaneously modeling MPXV infection and the resultant inflammatory response by integrating macrophages into organoids to establish macrophage-augmented organoids (MaugOs). Furthermore, reconstructed human skin (RHS) models developed by integrating primary human keratinocytes and stromal cells can be explored for studying MPXV infection and testing therapeutics in future research. The pattern was created using BioGDP.com (18)

Mpox, the disease caused by MPXV infection, is characterized by distinct clinical features and systemic manifestations (Fig. 1A). Mpox usually starts with flu-like symptoms, including fever, headache, muscle aches, and fatigue, followed by the emergence of a distinct rash (15). Skin lesions result from robust viral replication in keratinocytes, causing necrosis and fluid-filled blisters (16). The clinical presentation of mpox is variable among different populations, as demonstrated in the global outbreak and the current outbreaks in Africa, which may be attributed to the different clades, transmission dynamics, and level of immune competency (6, 17).

Across all clades, systemic symptoms and manifestations are common, and lymphadenopathy is a key feature, reflecting systemic inflammatory response (6). Mpox-associated systemic manifestations include cardiovascular and neurological complications, kidney injury, proctitis (inflammation of the rectum), respiratory symptoms, vision impairment, and pregnancy-related manifestations (2, 4, 19–23). These systemic manifestations are often associated with more severe clinical outcomes including fatality (2, 23, 24). Although the mechanisms underlying disseminated infection remain unknown (25), the virus likely infects lymphocytes and spreads via the bloodstream (23, 26), affecting the skin, gastrointestinal tract, liver, kidney, lung, brain, and reproductive organs, as well as causing viremia and multi-organ injury and inflammation (16, 27, 28).

### Vulnerable populations

The 2022–2023 global outbreak primarily affected men who have sex with men, with a substantial proportion having (advanced) HIV infection (29). Consistent with epidemiology in endemic countries prior to 2022, concurrent outbreaks in Africa affect the general population, but disproportionately threaten young children and pregnant women (30–34), as well as people living with HIV (35, 36). Those individuals are at the highest risk of complications and death, especially in resource-limited settings with poor access to healthcare (14, 37). In general, the case fatality rate is higher in clade 1 compared with clade 2 MPXV infection. Alarmingly, in DRC, children younger than 15 years accounted for 70% of reported mpox cases and 83% of fatalities between January and May 2024 (31). In a cohort study between September 2023 and June 2024 in DRC, 14 infected pregnant women were hospitalized, of whom 8 had fetal loss (24), and MPXV genome was detected in the placenta by PCR (23). Although only a small number of deaths were reported during the global outbreak, most of these cases were immunocompromised people with advanced HIV (29, 38). Solid organ transplant recipients infected with clade IIb MPXV also experienced severe outcomes, likely due to immunosuppressive medications (39). Furthermore, persistent MPXV infection with severe manifestations may occur in immunocompromised patients, especially those with advanced HIV (40, 41). A cohort study in Nigeria found that co-infection of MPXV with varicella zoster virus (VZV) is an independent risk factor for severe disease (42). With advancing age or immunosuppression, reactivation of latently infected VZV can be relatively common (43). Furthermore, secondary bacterial infections and sepsis can occur and may cause severe complications (42, 44).

This collectively highlights the urgent need for effective therapeutics for treating severe mpox, which in turn requires diverse pipelines for therapeutic discovery and robust experimental models to authentically replicate disease manifestations and test therapeutics.

## EXPERIMENTAL MODELS

### Conventional cell line models

Currently, *in vitro* experiments on MPXV research mainly rely on immortalized cell lines originating from monkey and human sources (Fig. 1B) (45). These cell lines generally support robust MPXV infection but fail to replicate key disease manifestations seen in mpox patients due to their intrinsic limitations, such as harboring enormous genetic, epigenetic, and functional alterations (46).

### Animal models

The mouse (*Mus musculus*) model has been widely employed to study host responses and to test vaccine and therapeutic candidates (Fig. 1B) (47–50). In addition, several studies have reported the use of other MPXV-permissive species such as prairie dogs (*Cynomys ludovicianus*), ground squirrels (*Sciuridae*), and rabbits (51–53). These animal models remain far from replicating human mpox disease, such as failing to present the formation of pustules, a key clinical feature. In contrast, non-human primates, including cynomolgus monkeys, rhesus macaques, marmosets, and baboons, are better in modeling mpox disease manifestations and progression and are thus useful tools for assessing vaccines and therapeutics (54–57). In an experimental study, cynomolgus monkeys infected with MPXV demonstrated systemic viral dissemination through a monocytic cell-associated viremia, resulting in lesions affecting the lymphoid tissues, skin, oral mucosa, gastrointestinal tract, liver, and reproductive system (58), replicating the clinicopathologic features observed in severe mpox. However, there are major ethical considerations for using these animals (59). Intriguingly, a very recent study suggests that the fire-footed rope squirrel (*Funisciurus pyrropus*), a forest-dwelling rodent in West and Central Africa, may be a reservoir for MPXV (60, 61). It would be interesting to explore whether this species can better model mpox disease.

### Organoids and other innovative models

Organoids are “mini-organs” cultured from tissue stem cells or induced pluripotent stem cells (iPSCs) (62). Compared to cell lines, human organoids are superior in replicating the architecture, organization, composition, diversity, and functionality of cell types of the original tissue or organ, while circumventing species barriers present in animal models (63, 64). Human iPSC (hiPSC)-derived skin organoids have proven to be an excellent model for MPXV infection and therapeutic testing (65, 66). To model systemic manifestations, hiPSC-derived kidney organoids (67), brain organoids (68), as well as neural progenitor cells (69) have been successfully employed for MPXV infection. Of note, a recent study using hiPSC-derived colon organoids failed to effectively model MPXV infection (70). Although the underlying explanation remains unknown, this model may lack the right cell types that are permissive to MPXV replication. In contrast, robust replication of MPXV has been established in primary organoids cultured from human small intestine and rectal tissues (71), as well as human liver tissue (72). Given the capability of rapid expansion in culture, these primary organoids enable small- to medium-scale drug screening (46, 71).

One remaining challenge of epithelial organoids is the lack of interactions with other cell types, making it difficult to accurately model the complexity of mpox disease. A recent study demonstrated the proof of concept by integrating macrophages into organoids, generating so-called macrophage-augmented organoids (MaugOs) (72, 73), to simultaneously model MPXV infection and the resultant inflammatory response. Further research should also consider other immune cell types (e.g., dendritic cells) as well as stromal cells to further leverage the organoid model for replicating the multi-layered pathophysiology of mpox, which would enable the testing of advanced therapeutic strategies. Reconstructed human skin (RHS) models by integrating primary human keratinocytes and stromal cells have been employed for studying skin diseases and testing the efficacy and safety of therapeutics (74). These skin models are being further advanced by integrating immune cells, in particular Langerhans cells, a key player in sensing skin infections (75). Integration of RHS into deendothelialized organ-on-chip platforms with flowing immune cells offers a more dynamic and physiologically relevant system (76). Such authentic human skin models warrant exploration for modeling mpox (Fig. 1B).

## THERAPEUTIC DEVELOPMENT

### Current status

#### Tecovirimat

**Fig 2:**
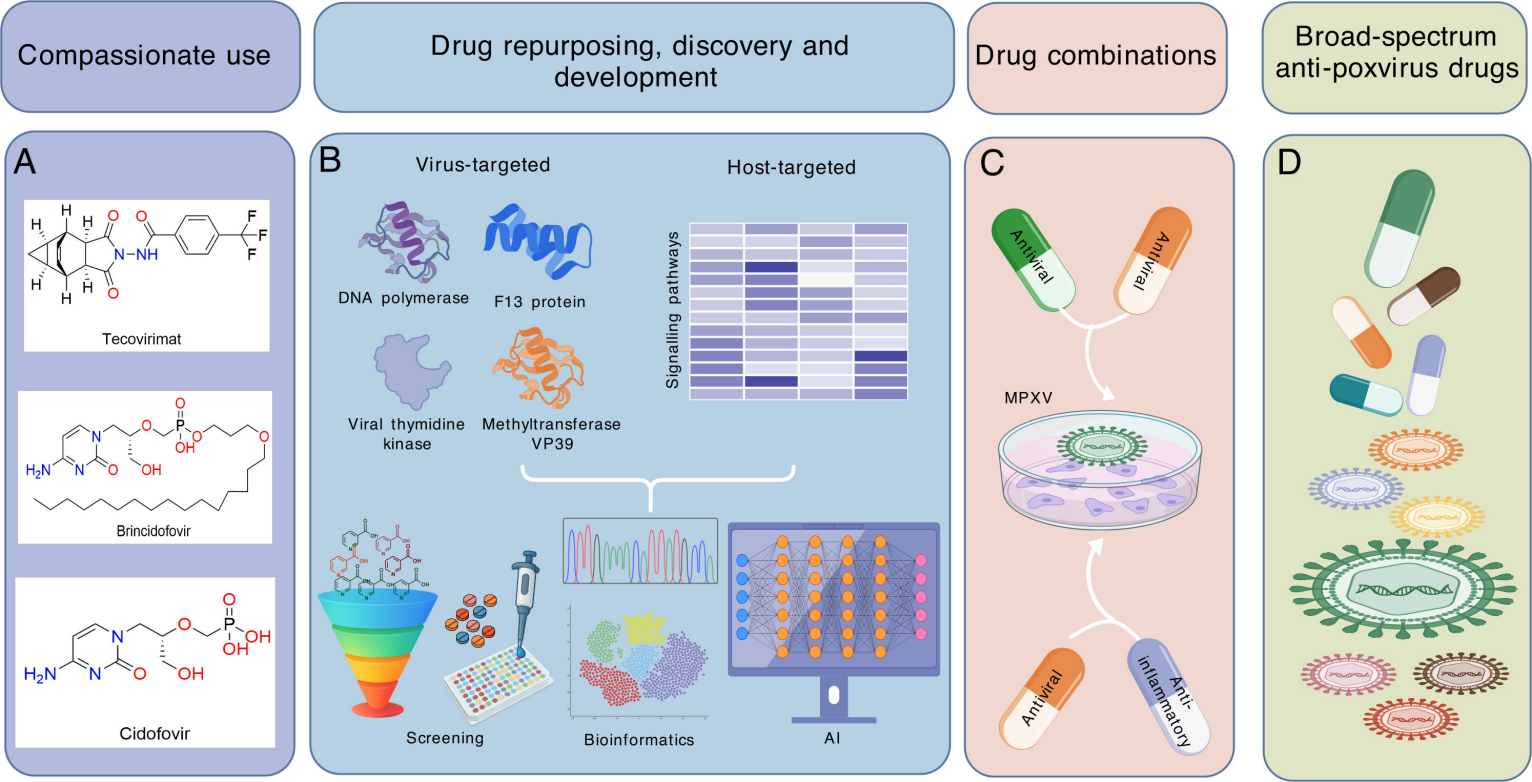
Therapeutic development for treating mpox. (**A**) Antiviral drugs prescribed for compassionate use in mpox. Tecovirimat, brincidofovir, and cidofovir are currently prescribed as compassionate use in treating mpox. However, results from two recent randomized, placebo-controlled trials showed that tecovirimat failed to benefit both clade I and clade II monkeypox virus (MPXV) infected patients. A randomized, double-blind, placebo-controlled trial is currently underway to evaluate the efficacy of brincidofovir in treating mpox. (**B**) Drug repurposing, discovery, and development for mpox therapeutics. MPXV encodes a large number of viral proteins and enzymes necessary for its life cycle, which serve as promising targets for therapeutic development. For example, in addition to viral DNA polymerase and F13 protein, viral thymidine kinase and methyltransferase VP39 can be explored for developing direct-acting antivirals. MPXV actively interacts with human host factors to drive infection and disease manifestations, making these host pathways important targets for developing host-directed therapies. Drug (virtual) screening, bioinformatics, and AI-driven techniques can be used to repurpose, discover, and develop new therapeutics. (**C**) Potential drug combination approaches for mpox treatment. Combinational antiviral therapies often can exhibit synergism and prevent the development of drug resistance. This highlights the potential of combining antiviral agents to achieve synergistic anti-MPXV activity and to prevent drug resistance. Severe mpox is often accompanied by pathological inflammation driven by immune cells, which exacerbates disease severity. Current antiviral therapies target the pathogen but do not address hyperinflammation. Therefore, combining antivirals with anti-inflammatory drugs should be investigated to simultaneously inhibit infection and control hyperinflammation. (**D**) Broad-spectrum anti-poxvirus drugs. In addition to MPXV, the Orthopoxvirus genus includes several other members, such as variola virus (which causes smallpox), vaccinia virus (used in the smallpox vaccine), as well as cowpox, rabbitpox, and camelpox viruses, which can cause spillover infections from animals to humans. Orthopoxviruses continue to pose an emerging threat to public health. Therefore, it is important to identify drug candidates with broad-spectrum antiviral activity to prepare for future poxvirus epidemics. The pattern was created using BioGDP.com (18).

Tecovirimat is an oral and intravenous formulated antiviral drug approved by the United States Food and Drug Administration (FDA) for treating smallpox (Fig. 2A) (77, 78). Its mechanism of action is conserved across different orthopoxviruses by disrupting the major envelope wrapping protein VP37 to prevent infectious virus production (79). It was widely prescribed as compassionate use for treating mpox during the global outbreak (80–83).

In various experimental models, tecovirimat has been shown to potently inhibit the infections of MPXV as well as related orthopoxviruses (Table 1) (66, 67, 77, 84–94). In striking contrast, two recent randomized, placebo-controlled trials showed no clinical benefit of oral tecovirimat treatment for patients infected with clade I (95) or clade II (96) MPXV strains (Table 1). This stark discrepancy observed between experimental models and clinical trials raises critical questions as to whether currently available experimental models are reliable in replicating mpox disease manifestations or are authentic for assessing therapeutics. We speculate that, by only preventing virus production but not any other steps of the viral lifecycle, tecovirimat may not be potent enough to completely eradicate the virus in infected hosts. Future research is urgently needed to clarify the mechanisms behind tecovirimat’s ineffectiveness under tested treatment protocols (95, 96). This knowledge will be critical for determining whether (or how) to pursue tecovirimat’s therapeutic potential further in clinical practice.

**TABLE 1:** Summary of clinically used antiviral drugs for treating mpox

	Tecovirimat	Cidofovir	Brincidofovir
FDA-approved indication	Smallpox (77)	Cytomegalovirus retinitis in patients with acquired immunodeficiency syndrome (97)	Smallpox (98)
Route(s) of administration	Oral; intravenous (78)	Intravenous (99)	Oral (99)
Mechanism of action	Disrupt the function of *Orthopoxvirus* VP37 envelope wrapping protein to prevent the production of infectious virus (79)	Inhibit DNA polymerase activity and act as a nucleotide analog to incorporate into viral DNA to interfere with synthesis (100)	Lipid-conjugated prodrug of cidofovir (101)
Preclinical findings of anti-MPXV activity	Inhibit MPXV infection in cell line and organoid models *in vitro* (66, 67, 84–86) Improve survival rates in animal models *in vivo* (e.g., nonhuman primates, macaques, mice, squirrels, and prairie dogs) (86, 88–94)	Inhibit MPXV infection in cell line models *in vitro* (102, 103) Exert antiviral efficacy in animal models *in vivo* (e.g. macaques) (104)	Inhibit MPXV infection in cell line models *in vitro* (103) Exert antiviral efficacy in animal models *in vivo* (e.g., prairie dogs and mice) (105, 106)
Compassionate emergency use for mpox	Widely used (80–82)	Rarely used (97)	Occasionally used (97, 107)
Clinical trials for mpox	The PALM007 trial (NCT05559099): Failed to reduce the duration of mpox lesions and the mortality rate among children and adults infected with clade I MPXV (95) The STOMP trial (NCT05534984): Failed to reduce the time to lesion resolution or pain among adults infected with clade II MPXV (96) UNITY trial: failed to reduce time to lesion resolution	Not available	The MOSA trial: A pan-African randomized platform adaptive trial for the mpox study to evaluate the safety and efficacy of different antivirals, either alone or in combination; first started by evaluating the safety and efficacy of brincidofovir for treating mpox in Africa (ongoing) (108)

#### Cidofovir and brincidofovir

Brincidofovir is a lipid-conjugated prodrug of cidofovir and is the second antiviral approved for treating smallpox under FDA’s Animal Rule (Fig. 2A) (98, 101). Cidofovir has poor bioavailability and must be administered intravenously, whereas brincidofovir has increased oral bioavailability (99). Cidofovir can inhibit DNA polymerase activity and act as a nucleotide analog to incorporate into viral DNA to interfere with synthesis (100). Cidofovir and brincidofovir have broad-spectrum antiviral (BSA) activities against many DNA viruses, and their anti-MPXV activities have been demonstrated in a wide range of experimental models (Table 1) (87, 97, 102–107). A randomized, double-blind, placebo-controlled trial is currently underway in Africa, mainly in the DRC, to assess the safety and efficacy of brincidofovir in treating mpox (Table 1) (108). The upcoming results of this study will be crucial in determining the potential role of brincidofovir in addressing the mpox crisis.

### Future perspectives

#### Drug repurposing

Current efforts on the development of therapeutics against mpox are largely empirical and siloed. Given that the most affected patients are mainly from vulnerable/marginalized populations and resource-limited settings, there is limited economic incentive for the pharmaceutical industry to develop new mpox therapeutics. Repurposing existing medications represents a realistic and attractive approach, and identified candidates lend themselves to expeditious clinical investigation (109). The total number of all approved drugs globally is about 4,700, of which about 3,000 are small-molecule drugs (110). Technically, it is highly feasible to screen these drugs in MPXV-infected cell line models. A recent study screened a library of 240 known BSAs in MPXV-infected primary human intestinal organoids (71). In addition to clinically approved medications, these screened BSAs extend to other proven safe-in-human agents, including those that have passed phase I clinical trials but are not yet clinically approved (111). The investigators identified a list of MPXV inhibitors more potent than the positive control cidofovir, including approved medications such as the anti-cancer drug clofarabine; the antiparasitic agent emetine; and investigational agents such as beclabuvir (for hepatitis C) and AVN-944 (anti-cancer) (71), although further validation is required before clinical testing.

#### Direct-acting antivirals

The MPXV genome contains roughly 193 open reading frames encoding viral proteins with over 60 amino-acid residues (112), offering numerous opportunities for antiviral targeting. Nucleoside analogs, such as cidofovir, are a major group of promising BSA candidates targeting viral DNA polymerase. It has been recommended to direct research efforts towards the optimization and development of cidofovir-related acyclic nucleoside phosphonates, with increased specificity and potency against poxvirus DNA polymerases (113). These efforts can be facilitated by recently available data extensively characterizing the MPXV DNA polymerase structure (114–116). In addition, many other viral proteins and enzymes, such as the viral thymidine kinase (117) and methyltransferase VP39 (118), can be explored for developing direct-acting antivirals.

#### Host-targeted antivirals

On the other hand, MPXV actively interacts with the human host, driving infection and disease manifestations. The host’s innate immunity acts as the first line of defense against MPXV infection (119). MPXV replicates in the cytoplasm, and innate immune cells such as monocytes, macrophages, and dendritic cells detect the infection through pattern recognition receptors (PRRs). It has been shown that the cytosolic PRRs, cyclic GMP-AMP synthase (cGAS), and stimulator of interferon genes (STING), can detect MPXV infection and trigger type I interferon (IFN) response (120). As the second line of defense, adaptive immunity plays an important role in controlling MPXV spread through producing neutralizing antibodies and recruiting cytotoxic immune cells to target virus-infected cells (121). Further research on host immune responses during MPXV infection would help to better understand vaccine responses but also to develop host-targeted therapeutic strategies. For example, treatment with clinically approved type I IFN (pegylated IFN alpha-2b) has been shown to effectively reduce MPXV viral titers and mpox disease severity in rhesus macaques (120).

Through computational analysis of the MPXV-human protein–protein interaction network, a study identified six candidate drugs for treating mpox through a drug repurposing approach (122), although experimental validation is required to confirm their antiviral activity. Another study employed multi-omics approaches to uncover key host factors and pathways as therapeutic targets and identified FDA-approved drugs, including kinase inhibitors and steroid hormone receptor agonists as potential MPXV inhibitors (123), but again, experimental validation is required to confirm these candidates. Unlike antivirals that directly act on viruses, these antiviral candidates, which modulate host cell factors that the viruses hijack to support their infection, are expected to have higher barriers to drug-resistance development (124).

#### Artificial intelligence (AI)-driven drug repurposing and discovery

AI-driven innovations offer transformative potential for drug repurposing and discovery (125). AI-driven approaches (e.g., machine learning [ML]/deep learning [DL]) are increasingly being used in drug development (126). These tools can be particularly powerful in drug discovery and repurposing in response to emerging epidemics, such as the ongoing mpox emergency. Conserved viral proteins or enzymes, such as viral replicases, can be prime targets for developing direct-acting antivirals. Crystal structures of viral proteins from newly surfaced viruses are typically not available, for example, until at least a year after their emergence. In the interim, AI-based structure prediction tools, such as AlphaFold developed by DeepMind and comparable models, can be used to accurately predict a protein’s 3D conformation directly from its amino acid sequence (127). Structure-based methods such as molecule docking and dynamics, or their ML/DL-based counterparts (128), can be utilized to assess the interaction-related potential of candidate molecules against the intended target protein structure. A recent study utilized an AI-guided virtual screening pipeline that integrates docking with molecular dynamics and identified existing antivirals (e.g., the HIV integrase inhibitor elvitegravir) as potential MPXV inhibitors (129). Apart from that, ligand-based (data-centric) bioactivity prediction methods can predict potential ligands of selected proteins using the existing experimental bioactivity data as the model’s training data set, without requiring the target proteins’ structure. A representative example is DEEPScreen, a deep convolutional network that learns from 2D compound representations to predict interactions across 704 widely studied protein targets, demonstrating high predictive accuracy (130). However, like crystal structures of viral proteins, bioactivity data are also usually not available at the beginning of epidemics. Given that any emerging viral pathogen would likely share some common features with particular existing viruses, ML/DL-based models could then be trained by curating data sets of existing experimental bioactivity data derived from highly similar or related viruses. Therefore, these trained models can be deployed to predict compounds or repurpose existing drugs to target emerging viral pathogens.

AI-driven *de novo* molecular generation provides a compelling alternative when virtual screening of approved drugs and drug-like libraries fails to produce suitable candidates. In this field, transformer-based generative models—architecturally akin to large language models such as GPT—are trained on vast corpora of chemical structures and consequently learn the statistical distribution of drug-relevant chemical space. Either during training or at inference, these models can be conditioned on user-specified objectives, such as high binding affinity to a designated protein (131) or adherence to predefined physicochemical and drug-likeness-related properties (132).

In parallel, further AI-driven advancements in bioinformatics and computational biology, for example, deep interactome learning for designing new drug candidates (133), can be explored to identify host-targeted antivirals at scale (122). After lead optimization, drug discovery enters the pre-clinical and clinical phases, the most capital-intensive and failure-prone stages of development. To reduce attrition, recent ML/DL models have been built to predict the likelihood that a candidate will achieve regulatory approval, drawing on data from completed trials—especially Phase II and Phase III—and on compound-specific features (134). These AI-based innovative approaches constitute an unprecedented opportunity for drug discovery and repurposing to tackle the mpox crisis (Fig. 2B).

#### Antiviral drug combinations

Despite demonstrated efficacy in the early stages of drug development, antiviral monotherapies are often suboptimal in clinical settings, whereas combination therapies are being increasingly explored for treating viral infections in clinical practice (135). Combination therapies often exhibit synergism and could achieve similar efficacy to individual components while requiring lower dosage, with potentially fewer and milder adverse effects (136). Importantly, antiviral combinations can better prevent the development of drug resistance, which has emerged as a critical issue for tecovirimat treatment in mpox patients (29)—a potential issue for cidofovir and brincidofovir as well (137). Since persistent MPXV infection may occur in some immunocompromised patients (138, 139), prolonged treatment with antiviral monotherapy could increase the risk of drug resistance. Furthermore, host factors such as the apolipoprotein B editing complex (APOBEC3) cytosine deaminase have been recognized to catalyze single base pair mutations to drive MPXV evolution (140). It is an intriguing question whether this mechanism of mutagenesis would contribute to pre-existing drug resistance or accelerate its development.

The design of antiviral drug combinations should consider their complementary mechanisms of action. In this respect, we foresee the potential for combining tecovirimat with brincidofovir to achieve synergistic anti-MPXV activity and to prevent drug resistance (141). Once the development landscape of mpox therapeutics expands and more drug candidates are identified, innovative approaches, including the use of AI, could be employed to design drug combination regimens (136). This could incorporate multi-dimensional data including potency of single drug treatment, toxicity, conserved druggable virus-host interactions, mechanisms of action, immunomodulatory properties, and drug-drug interactions. Such algorithms should enable the prediction of synergistic, additive, and antagonistic antiviral activities, prioritizing optimal drug combinations, and eventually reducing experimental costs and translational time (Fig. 2C).

#### Antiviral and anti-inflammatory drug combination

Severe mpox may be accompanied by pathological inflammation driven by immune cells, which exacerbates disease severity (142). Current antiviral therapies target the pathogen, but elimination of the virus is not in itself sufficient to control hyperinflammation. Although existing anti-inflammatory drugs may provide the desired therapeutic benefits, a common side effect is to facilitate host conditions that support infection (143). For example, classic anti-inflammatory steroids such as dexamethasone have been shown to improve clinical outcomes of severe COVID-19 that are linked to inhibition of inflammatory responses (144). However, these drugs have been shown to exert direct pro-viral effects of SARS-CoV-2 infection in animal models (145).

An mpox patient who developed neurological complications with extensive inflammatory involvement of the brain and spinal cord was managed with steroids without specific antiviral treatment (146). Another case report has suggested that steroid treatment may help manage mpox-associated inflammatory syndromes (147). However, we foresee that effective treatment for severe mpox requires simultaneous inhibition of both viral replication and hyperinflammation, presumably through antiviral and anti-inflammatory drug combinations (Fig. 2C).

#### Preparedness for poxvirus epidemics

In addition to MPXV, the Orthopoxvirus genus consists of several other members, including variola virus that causes the deadly smallpox, vaccinia virus that is used in the smallpox vaccine, as well as cowpox, rabbitpox, and camelpox viruses. Historically, smallpox is estimated to have killed up to 300 million people in the 20th century and around 500 million people in the last 100 years of its existence (148). The human population is an ecological niche for orthopoxviruses (25). Spillover infections from animal hosts to humans have been occupationally reported for cowpox, although rarely for rabbitpox and camelpox viruses. Spillover infection of cowpox virus is relatively common in Europe, which can cause severe complications in infected patients (149). Zoonoses caused by vaccinia virus are also common in South America, attributed to its establishment in rodent populations during the smallpox eradication campaign (25). Borealpox virus (formerly known as the Alaskapox virus) has recently been reported to cause a fatal infection in an immunosuppressed patient in the United States (150).

Following smallpox eradication in 1980, the public was no longer required to be routinely vaccinated against the disease. This is postulated as one of the key factors contributing to the emergence of subsequent mpox epidemics and sporadic poxvirus zoonoses (151). Thus, orthopoxviruses inevitably pose an emerging threat to global public health. Furthermore, the failure of tecovirimat for treating mpox in rigorous clinical trials raises serious concerns regarding the validity of stockpiling this drug for smallpox or mpox preparedness (152). Therefore, to strengthen preparedness for future poxvirus epidemics, we propose a pan-poxvirus approach to identify drug candidates with BSA activity. Technically, it is highly feasible to develop both virus-targeted and host-directed pan-poxvirus antiviral drugs, given the similarities of these viruses and the shared host machineries for infection (Fig. 2D) (153).

## CONCLUDING REMARKS

The rapidly evolving epidemiological and clinical characteristics of mpox highlight the significant challenges in controlling this re-emerging viral disease. Distinct MPXV clades are adapting and spreading beyond traditional endemic regions, increasingly affecting diverse and vulnerable populations, particularly in resource-limited endemic settings. The WHO recommends PCR-based tests to detect viral DNA for diagnosing mpox and has approved several diagnostic tests for emergency use (154). However, these tests are constrained by the need for laboratory equipment and trained personnel. There is an urgent need for developing point-of-care tests to respond to the current outbreak in Africa and to strengthen preparedness for future outbreaks (155). Although the availability of mpox vaccines is increasing in Africa, significant challenges persist, including insufficient vaccine doses, unequal access, and limited healthcare infrastructure (156).

Recent outbreaks have underscored the urgent need for effective therapeutics, alongside vaccines, to mount an adequate response to the mpox emergency. Suboptimal clinical efficacy observed with current antivirals, especially the failure of tecovirimat monotherapy (152, 157, 158), further emphasizes the limitations of existing treatment strategies and the pressing need for continued drug development. Addressing these gaps requires a deeper understanding of the shifting epidemiology and clinical spectrum of mpox, as well as the creation of robust experimental models that more accurately reflect human disease. While advances such as human organoids have provided innovative platforms for studying MPXV infection and evaluating antiviral agents, these models still fall short of fully capturing the complexity of mpox pathogenesis. Moving forward, fostering innovations in experimental model development, drug repurposing, and new therapeutic discovery will be essential to mitigate the impact of ongoing mpox outbreaks and to strengthen preparedness for future poxvirus epidemics.
